# Lower Methylation of the *ANGPTL2* Gene in Leukocytes from Post-Acute Coronary Syndrome Patients

**DOI:** 10.1371/journal.pone.0153920

**Published:** 2016-04-21

**Authors:** Albert Nguyen, Maya Mamarbachi, Valérie Turcot, Samuel Lessard, Carol Yu, Xiaoyan Luo, Julie Lalongé, Doug Hayami, Mathieu Gayda, Martin Juneau, Nathalie Thorin-Trescases, Guillaume Lettre, Anil Nigam, Eric Thorin

**Affiliations:** 1 Montreal Heart Institute, Research Center, Université de Montréal, Montreal, Quebec, Canada; 2 Department of Pharmacology, Faculty of Medicine, Université de Montréal, Montreal, Quebec, Canada; 3 Cardiac Rehabilitation and Prevention Center (EPIC) of the Montreal Heart Institute, Université de Montréal, Montreal, Quebec, Canada; 4 Department of Medicine, Faculty of Medicine, Université de Montréal, Montreal, Quebec, Canada; 5 Department of Surgery, Faculty of Medicine, Université de Montréal, Montreal, Quebec, Canada; Goethe University, GERMANY

## Abstract

DNA methylation is believed to regulate gene expression during adulthood in response to the constant changes in environment. The methylome is therefore proposed to be a biomarker of health through age. ANGPTL2 is a circulating pro-inflammatory protein that increases with age and prematurely in patients with coronary artery diseases; integrating the methylation pattern of the promoter may help differentiate age- *vs*. disease-related change in its expression. We believe that in a pro-inflammatory environment, *ANGPTL2* is differentially methylated, regulating ANGPTL2 expression. To test this hypothesis we investigated the changes in promoter methylation of *ANGPTL2* gene in leukocytes from patients suffering from post-acute coronary syndrome (ACS). DNA was extracted from circulating leukocytes of post-ACS patients with cardiovascular risk factors and from healthy young and age-matched controls. Methylation sites (CpGs) found in the *ANGPTL2* gene were targeted for specific DNA methylation quantification. The functionality of *ANGPTL2* methylation was assessed by an *in vitro* luciferase assay. In post-ACS patients, C-reactive protein and ANGPTL2 circulating levels increased significantly when compared to healthy controls. Decreased methylation of specific CpGs were found in the promoter of *ANGPTL2* and allowed to discriminate age *vs*. disease associated methylation. *In vitro* DNA methylation of specific CpG lead to inhibition of *ANGPTL2* promoter activity. Reduced leukocyte DNA methylation in the promoter region of *ANGPTL2* is associated with the pro-inflammatory environment that characterizes patients with post-ACS differently from age-matched healthy controls. Methylation of different CpGs in *ANGPTL2* gene may prove to be a reliable biomarker of coronary disease.

## Introduction

Cardiovascular diseases (CVD) are known to be caused by the prolonged exposure to a growing list of risk factors such as tobacco use, unhealthy diet, physical inactivity, obesity, hypertension, dyslipidemia and metabolic disorders [1, 2]. CVD are characterized by a state of low-grade chronic inflammation through the increased production of pro-inflammatory mediators [3].

Angiopoietin-like 2 (ANGPTL2) is a circulating protein with pro-inflammatory properties [4–8], which levels increase with aging in the general population [6]. The early involvement of ANGPTL2 in the pathogenesis of chronic inflammatory diseases in humans is supported by the elevated plasma ANGPTL2 concentration detected in patients suffering from CVD [4–6, 9], diabetes [5, 10, 11] and obesity [5, 12, 13] alongside other classical markers of inflammation such as C-reactive protein (CRP) [14, 15]; a positive correlation between serum CRP and ANGPTL2 has previously been reported in diabetic patients [5]. In line with these previous findings, recent studies propose that plasma ANGPTL2 is a promising biomarker for inflammatory diseases such as various cancers [16–19], atherosclerosis [5, 20], diabetes [5] and heart failure [21].

The origin of circulating ANGPTL2 is however problematic. Early reports state that ANGPTL2 is mainly produced from the adipose tissue [5], but its mRNA can also be detected in other organs [22] such as the skeletal muscle, heart [5] and endothelial cells [4]. Therefore, ANGPTL2 likely has both systemic and tissue-specific activities depending if it is secreted or expressed locally. ANGPTL2 has also been found to be expressed in mouse bone marrow derived macrophages [23], infiltrating mouse [24] and human macrophages [6, 24], as well as *in vitro*, in human primary peritoneal macrophages (RAW264.7) [25] and macrophage-like cell line (THP-1) [26]. Therefore, although ANGPTL2 could be used as a biomarker of inflammation like CRP, it is unlikely that ANGPTL2 is associated with a specific disorder. A more refined parameter characterizing ANGPTL2 would, therefore, provide more information of the health status of patients.

In this regards, it is well established that aging [27] and environmental stimuli, including risk factors for CVD [28], induce epigenetic changes such as DNA methylation that modify gene expression. The consequences of DNA methylation on gene transcription vary with their locations within the gene and they are highly specific of a cell type [29, 30]. In general, methylation of the promoter region has been shown to decrease gene expression [31], while in the gene body, methylation can induce up or down regulation of the expression [32, 33]. In mammalian cells, methylation is predominantly found on cytosines preceding a guanine called CpG dinucleotide. *ANGPTL2* has been shown to be increasingly methylated in ovarian cancer [34] and myelodysplastic syndrome [35], while *ANGPTL2* promoter methylation is decreased in osteosarcoma [36]. Taken together, these studies reveal a potential role of DNA methylation in *ANGPTL2* expression. *ANGPTL2* methylation has not been studied in CVD, despite considerable evidence now showing that DNA methylation is associated with inflammation [37–39] and atherosclerosis [28, 40]. CVD are associated with both global [41] and gene-specific [40, 42, 43] differentiated methylation profiles, notably in leukocytes. These epigenetic changes are also linked to known CVD risk factors such as smoking [44–46], hypertension [47, 48] and obesity [49, 50]. Hence, blood DNA methylation quantification is emerging as a powerful diagnostic tool that has been shown to predict all-cause mortality [51].

The aim of our project was to test whether *ANGPTL2* methylation in circulating leukocytes isolated from patients with a recent first cardiovascular event could identify differential methylation marks compared to age-matched healthy volunteers.

## Materials and Methods

### Participants

Fasting blood samples were collected from 33 patients (26 men / 7 women; 62±2 y) with post-acute coronary syndrome (ACS) who provided written informed consent and were recruited at the cardiovascular prevention center of the Montreal Heart Institute. Consecutive cases of post-ACS patients were recruited from September 2011 to December 2013 at the Montreal Heart Institute. Per day, an average of 3 to 4 patients was studied: 750 patients per year (3 patients x 250 days of recruitment), i.e 1500 cases within 2 years, were evaluated. Among those cases, only 2–3 patients per week were eligible, and at the end 46 patients were enrolled. Among these 46 eligible patients, 9 dropped (5 patients stopped the training program that they were supposed to follow during the study, 1 patient was already involved in another clinical study, 1 was unfit for the physical training, 1 developed *de novo* atrial fibrillation, 1 developed a new ACS during the study). Among the remaining 37 patients, blood was available for ANGPTL2 quantification only in 33 patients. The mean duration after ACS was 65±7 days (median of 51 days [25–249]). One patient was enrolled after a period >4 months, 249 days after the ACS. The study was approved by the Ethical Board of the Montreal Heart Institute. Post-ACS patients were hypertensive, diabetic, dyslipidemic, obese, smokers (Tables 1 and 2), and were new members of the cardiovascular prevention center. Baseline characteristics, comorbidities and the medication of the patients are presented in Table 1.

**Table 1:** Baseline parameters of post-ACS patients.

	Post-ACS patients (n = 33)
**Age (years)**	62±2
**Men**	26 (79%)
**Family history**	16/33 (49%)
**Actual Percutaneous transluminal coronary angioplasty**	33/33 (100%)
**Actual Myocardial infarction**	29/33 (88%)
**Actual Unstable angina**	4/33 (21%)
**Hypertension**	21/33 (64%)
**Type II diabetes**	4/33 (12%)
**Dyslipidemia**	27/33(82%)
**Obesity**	21/33 (64%)
**Smoking**	5/33 (15%)
**Ex smoking**	19/33 (58%)
**Medication**	
Statins	32/33 (97%)
Aspirin	32/33 (97%)
β-blockers	28/33 (85%)
Angiotensin Converting Enzyme inhibitors	26/33 (79%)
Clopidogrel/Pasugrel	24/33 (73%)
Nitrates	14/33 (42%)
Calcium channel blockers	2/33 (6%)
Angiotensin II receptor antagonists	3/33 (9%)

Data are mean ± SEM of n participants.

**Table 2:** Anthropometric, hemodynamic and metabolic parameters of participants.

	Young healthy controls	n	Age-matched healthy controls	n	Post-ACS patients	n
**Age (years)**	28±1	20	61±2 (20)	20	62±2	33
**Men**	5		4		26	
**VO**_**2**_**max (ml/min/kg LBM)**	54.8±2.4	20	44.6±2.0	20	29.7±1.0 *, a	32
**BMI (kg/m**^**2**^**)**	21.6±0.4	20	23.8±0.5	20	28.1±0.8 *, a	33
**Body fat (%)**	17.2±1.5	20	25.2±1.6 *	20	28.2±1.2 *	32
**SAP (mm Hg)**	112±3	19	118±3	20	122±3 *	33
**DAP (mm Hg)**	68±2	19	73±1	20	69±1	33
**Heart rate (bpm)**	67±2	20	65±2	20	65±2	33
**Glucose (mM)**	4.8±0.1	19	5.0±0.1	19	5.4±0.1 *, a	33
**Insulin (pM)**	34.7±3.3	19	38.4±3.0	17	79.2±9.4 *, a	33
**TG (mM)**	0.71±0.07	20	1.04±0.13	19	1.09±0.07 *	33
**Total Cholesterol (mM)**	4.2±0.1	20	4.8±0.2 *	19	3.0±0.1 *, a	33
**LDL (mM)**	2.3±0.1	20	2.9±0.2 *	17	1.5±0.1 *, a	33
**HDL (mM)**	1.6±0.1	20	1.6±0.1	18	1.0±0.1 *, a	33
**CRP (mg/L)**	0.89±0.25	20	0.99±0.20	15	2.20±0.46 *	31

Data are mean ± SEM of (n) participants.

*: p<0.05 versus Young healthy controls,

^**a**^: p<0.05 versus Age-matched controls (Kruskal-Wallis test).

BMI, Body mass index; SAP, Systolic arterial pressure; DAP, Diastolic arterial pressure; TG, Triglycerides; LDL, Low-density lipoprotein; HDL, High-density lipoprotein; CRP, C-reactive protein.

Blood samples were collected in EDTA and heparin tubes from post-ACS patients and from 20 young (28±1 years) healthy and 20 age-matched (61±2 years) healthy volunteers recruited in a previous study [20]. Control healthy volunteers had no comorbidities and no medication; baseline characteristics, inclusion and exclusion criteria for these healthy volunteers have been previously reported [20].

Inclusion criteria for the post-ACS patients were the following: 1) men or women aged ≥18 years; 2) with previous ACS (unstable angina, or non-ST elevation myocardial infarction (NSTEMI), or ST elevation myocardial infarction (STEMI) with the presence of 2/3 criteria *i*.*e*. typical chest pain, electrocardiographic ischemic change, or elevated troponin T; 3) complete revascularization defined as no major epicardial coronary artery or bypass graft with a residual diameter stenosis > 50% and no residual left main stenosis > 40%; 4) left ventricular ejection fraction > 40%; 5) stable doses of medication during the 4 weeks prior to enrolment (STEMI patients must be on a stable dose of β-blocker); 6) able to perform a maximal cardiopulmonary exercise test; 7) capacity and willingness to sign informed consent.

Exclusion criteria for the post-ACS patients were: 1) recent (< 6 months) coronary bypass surgery; 2) incomplete revascularisation; 3) left ventricular ejection fraction (LVEF) < 40%; 4) significant valvular heart disease defined as mitral stenosis, grade III-IV mitral insufficiency, moderate-severe aortic stenosis, moderate-severe aortic insufficiency; 5) uncontrolled hypertension defined as blood pressure >180/110 mmHg; 6) significant resting ECG abnormalities including left bundle branch block, non-specific intraventricular conduction delay, left ventricular hypertrophy and resting ST-segment depression; 7) chronic atrial fibrillation; 8) pacemaker or implantable cardioverter defibrillator; 9) low functional capacity on baseline maximal exercise test (<5 METs); 10) any contra-indication to exercise training or any condition limiting effort to a greater degree than the CAD (such as neurologic disease, peripheral artery disease, osteoarthritis). The information concerning the presence of inflammatory disorders is not available.

The anthropometric, hemodynamic and metabolic parameters of the controls and the post-ACS patients are summarized in Table 2. The research protocol was approved by the Research Ethics and New Technology Development Committee of the Montreal Heart Institute. Following collection, ice-blood samples were centrifuged at 4°C and plasma was stored at -80°C. ANGPTL2 concentration was quantified by a commercial enzymatic immunoassay kit, as previously described [20].

### DNA extraction and bisulfite conversion

**Fig 1:**
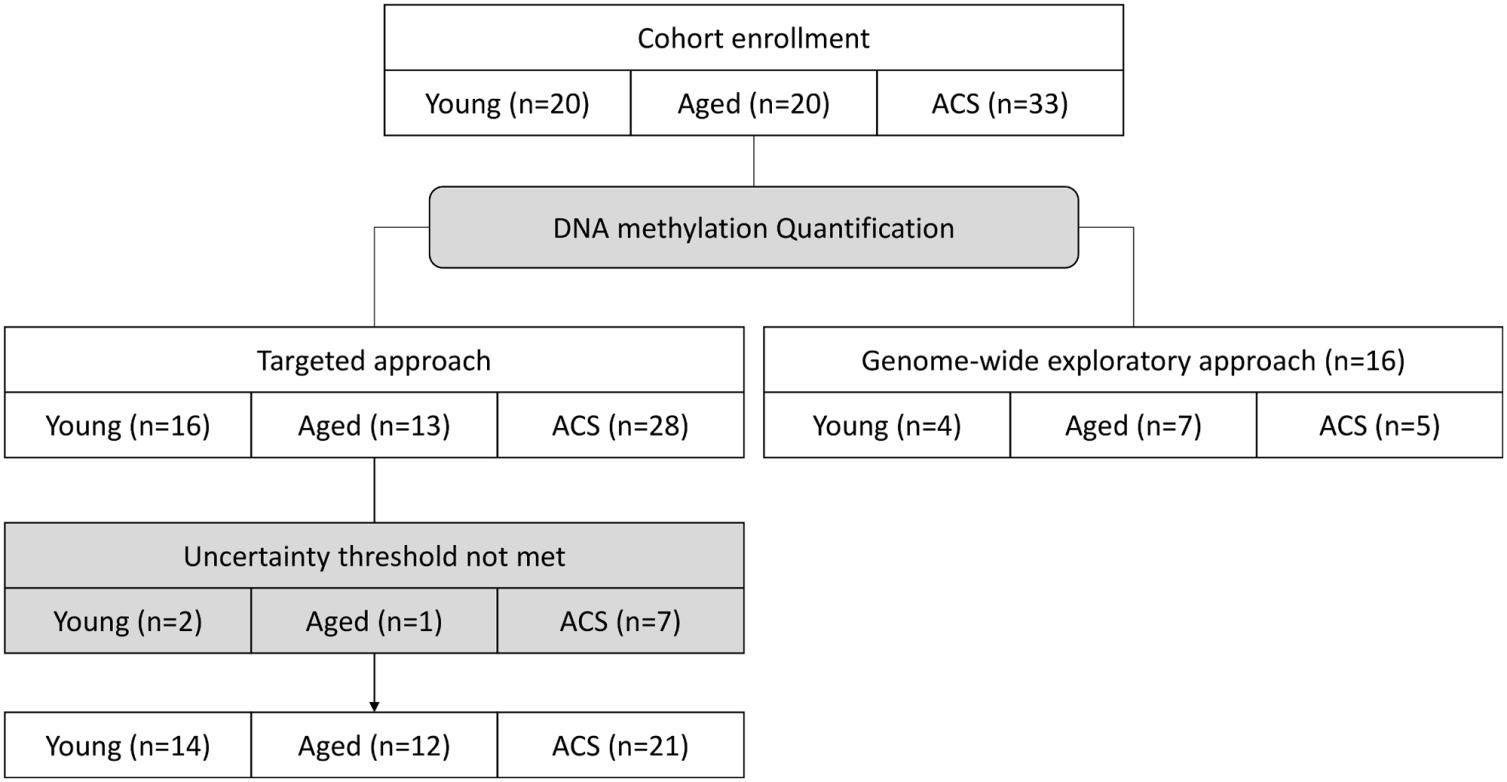
Flowchart illustrating the "n" values between groups throughout the study.

In all available samples (n = 20 young healthy controls, n = 20 age-matched healthy controls and n = 33 post-ACS patients) (Fig 1), total DNA was isolated from whole blood using a Qiagen DNeasy Blood & tissue kit following the manufacturer’s instructions. No selection of white blood cells was done, DNA was isolated from the whole white blood cells population. After extraction, DNA was quantified by NanoDrop (Thermo Scientific NanoDrop products, Wilmington, DE). DNA was then converted by bisulfite reaction using the EZ DNA Methylation-Gold kit (Zymo Research, Irvine, CA) following the manufacturer’s protocol.

### DNA methylation quantification

A genome-wide exploratory DNA methylation quantification protocol assay was performed on bisulfite-converted DNA using the Infinium Human Methylation 450 BeadChip Kit (Illumina, San Diego, CA) to obtain the methylation status of >485 000 CpG sites across the genome, as previously described [52]. This exploratory approach was performed in a small number of subjects (n = 4 young healthy controls, n = 7 aged healthy controls and n = 5 post-ACS patients) (Fig 1). We normalized probe intensities using the ARRm software [Fortin JP, Greenwood CMT, Labbe A: ARRmNormalization: Adaptive Robust Regression normalization for Illumina methylation data. In R package 1.0.0 edition; 2013.]. We removed probes that target a genomic region containing SNPs based on dbSNP version 137 (N = 82,694).

After this genome-wide exploratory DNA methylation quantification, fine mapping DNA methylation was then quantified by EpiTYPER assay (Sequenom, San Diego, CA), as previously described [53]. Gene-specific primers required for the assay are presented in Table 3. These experiments were performed at the McGill University and Génome Québec Innovation Centre, Montréal, Canada. This targeted approach was performed in all the remaining patients (n = 16 young healthy controls, n = 13 aged healthy controls and n = 28 post-ACS patients) (Fig 1). During the targeted approach, some samples showed undetectable methylation ratios because the maximum level of uncertainty was not met. In other words, any data with an estimated error larger than the uncertainty threshold (which is a value for the maximum amount of error accepted) was excluded and not displayed: some undetectable data were excluded in n = 2 young healthy controls, n = 1 aged healthy controls and n = 7 post-ACS patients. The fine targeted DNA methylation mapping was therefore performed in a total of n = 14 young healthy controls, n = 12 aged healthy controls and n = 21 post-ACS patients (Fig 1).

**Table 3:** EpiTYPER primer sequences. ANGPTL2-specific bisulfite primers required for PCR amplification.

Primer	Sequence
Forward	aggaagagagTTTATTTTTAAATTTTGGGGAAAGG
Reverse	cagtaatacgactcactatagggagaaggctCTCCAAAATCCTAAACTCAATTCAA

### Cloning of pCpG free-*ANGPTL2* vector

Constructions were done using the CpG free plasmid pCpGfree-promoter (Invivogen, San Diego, CA) as the backbone to study enhancer methylation. The *ANGPTL2* CpG region was amplified using forward 5’-TAAGCTCCTTCCCACGTGACCTCACAGAGTCG-3’ and reverse 5’-GATCCGACTCTGTGAGGTCACGTGGGAAGGAGCTTATGCA-3’ primers and subsequently inserted in the backbone using NsiI and BamHI restriction sites as previously described [54, 55].

### *In vitro* methylation, transient transfection and luciferase assay

Cloned vectors were isolated by Qiagen QIAprep Spin Miniprep kit (Qiagen). M. SssI CpG methyltransferase (New England Biolabs, Frankfurt, Germany) was used for *in vitro* methylation according to manufacturer’s instructions. Methylated DNA was then purified using the QIAquick gel extraction kit (Qiagen) and quantified by NanoDrop (Thermo Scientific NanoDrop products, Wilmington, DE). Methylation was confirmed by digestion with the methylation-sensitive restriction enzymes HhaI and HpaII. HEK293 cells grown to confluence on 96-well plates were transfected with the pCpG free-Gpx1 vector using Lipofectamine 2000 (Invitrogen). Twenty-four hours after transfection, luciferase activity was measured with the QUANTI-Luc reagent (Invivogen, San Diego, CA) by luminescence detection. Promoter activity was normalized to the total amount of protein measured by a Bradford assay (Biorad, Hercules, CA) [54, 55].

### Statistical Analysis

Results are presented as mean±SEM of (n) participants. One-way ANOVA (with Bonferonni post-test) or Kruskal-Wallis test (with Dunn post-test), unpaired t-test or Mann Whitney test were used where applicable, depending on Gaussian distributions, to test the difference between groups (Graph Pad Prism). A p-value of p<0.05 was considered statistically significant.

## Results

### Increased circulating ANGPTL2 concentration in post-ACS patients

**Fig 2:**
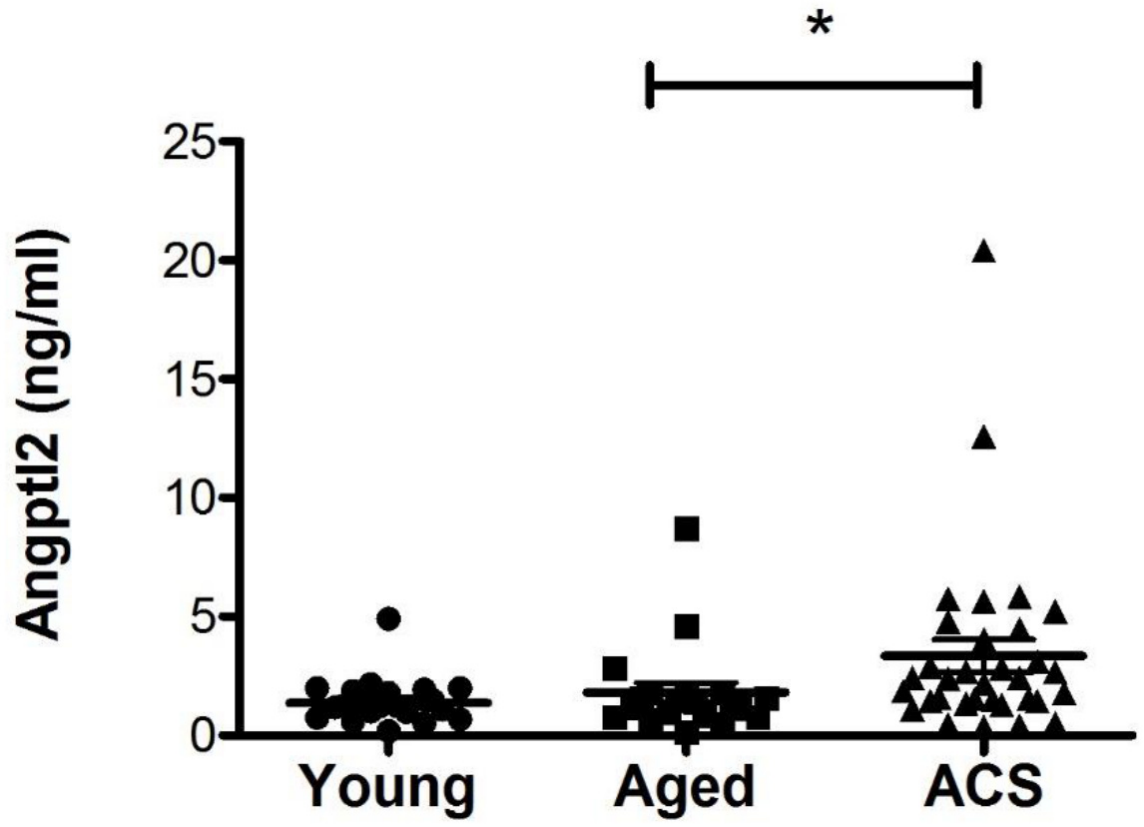
Increased ANGPTL2 in post-ACS patients. Fasting ANGPTL2 levels in the plasma of patients with post-acute coronary syndrome (ACS) (n = 33) compared to age-matched (n = 20) and young (n = 20) healthy controls. Data are mean ± SEM of (n) participants, *: p<0.05 *vs* Age-matched controls (Kruskal-Wallis test).

In accordance with our previously published data [4, 20], plasma ANGPTL2 concentration was higher in post-ACS patients (3.35 ± 0.67 ng/mL, n = 33) when compared to age-matched controls (1.80 ± 0.42 ng/mL, n = 20) (Fig 2). The post-ACS patients also display higher circulating CRP levels (2.20 ± 0.46 mg/L, n = 31) in comparison to young healthy controls (0.89 ± 0.25 mg/L, n = 20) (Table 2), illustrating the presence of a pro-inflammatory environment in these patients with various CVD risk factors (Table 1).

### Exploratory discovery of *ANGPTL2* methylation sites

**Fig 3:**
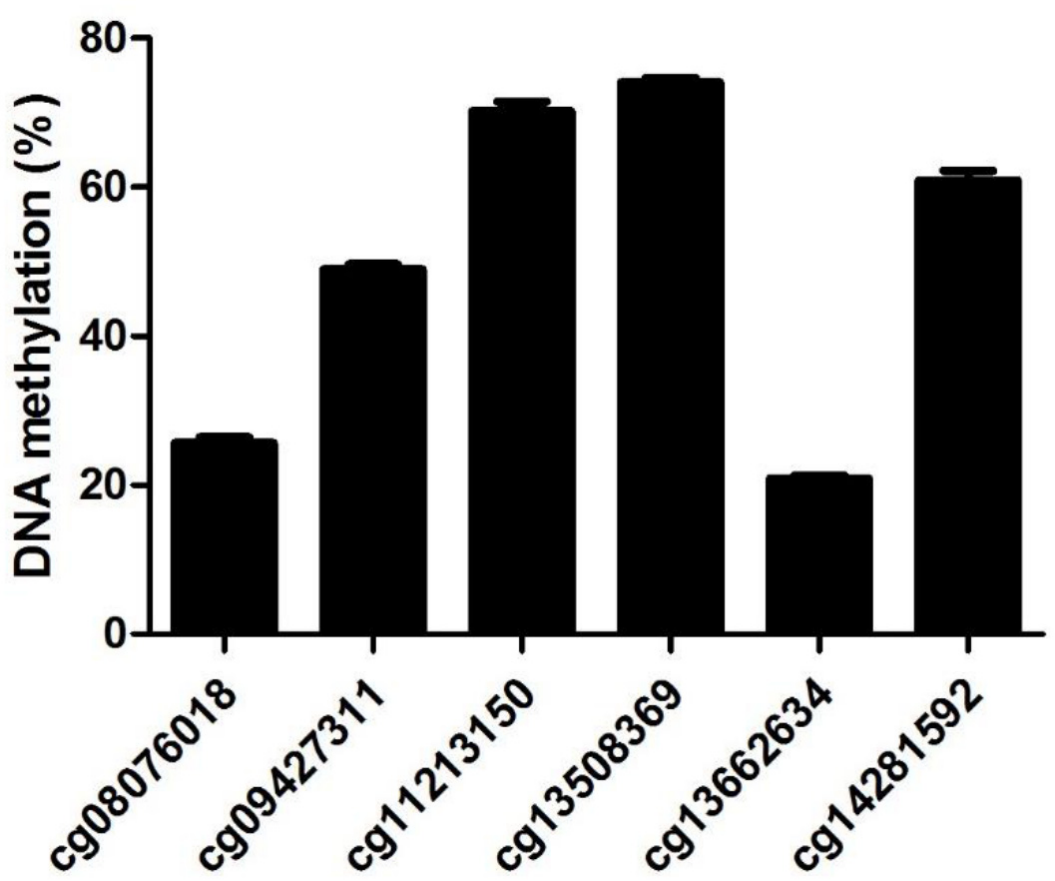
Detectable *ANGPTL2* methylation profile. Quantification of various methylation sites located in the *ANGPTL2* gene identified following a preliminary genome-wide exploratory assay. DNA samples were pooled from a small number of participants taken from all three groups. Data are mean ± SEM of a total of 16 patients from the young controls (n = 4), age-matched controls (n = 7) and post-ACS patients (n = 5).

We selected a total of 16 patients from the young controls (n = 4), age-matched controls (n = 7) and post-ACS patients (n = 5) for this genome-wide exploratory DNA methylation analysis. Out of the >485 000 probes included in the genome-wide quantification, only 6 probes were associated with the *ANGPTL2* gene: 1 probe (cg09427311) was distributed in the promoter region of the *ANGPTL2* gene, 2 probes (cg08076018 and cg13662634) were distributed in the 5’ region transcription start site and 3 probes were located in the gene body (cg11213150, cg14281592 and cg13508369) (Table 4). Significant levels of DNA methylation were detected in all 6 probes (Fig 3). However, due to the low number of individuals among each group, no statistical difference was observed between controls and post-ACS groups (data not shown).

**Table 4:** Exploratory probe coordinates. Genomic localisation of probes covering ANGPTL2 CpG sites analyzed by the Infinium HumanMethylation450 exploratory assay, as provided by the manufacturer.

Probe ID	Coordinates
cg08076018	chr9:128924901
cg09427311	chr9:128925551
cg11213150	chr9:128924278
cg13508369	chr9:128923847
cg13662634	chr9:128924769
cg14281592	chr9:128924134

Fine mapping DNA methylation was then quantified by EpiTYPER assay in larger groups; we chose to proceed with the investigation of probe cg09427311, since it is the only one located in the promoter region of *ANGPTL2* gene and that it is sufficiently far from the other probes to allow targeting with specific primers for downstream fine mapping analysis.

### Post-ACS patients have decreased ANGPTL2 methylation

**Fig 4:**
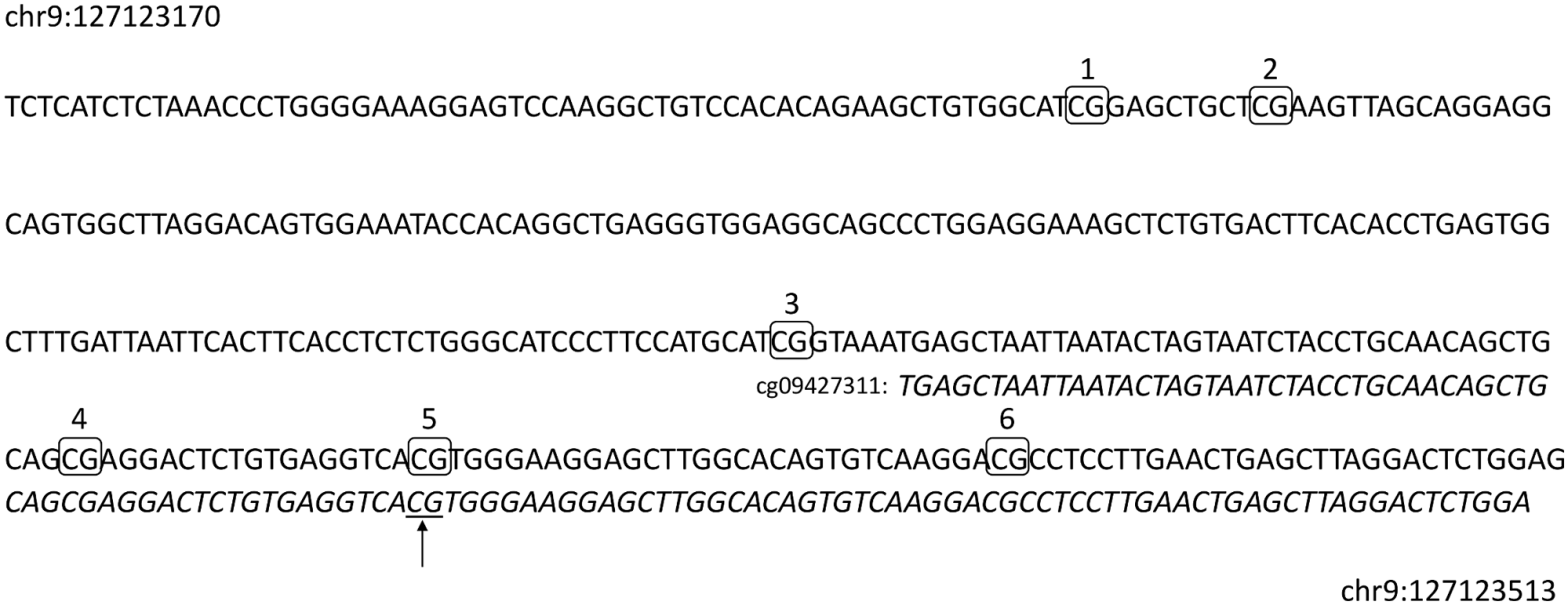
Fine mapping of *ANGPTL2* methylation profile. Identification (CpG1-6) and localization of CpGs targeted for DNA methylation quantification. The arrow represents the CpG previously characterized by probe cg09427311 during the exploratory analysis.

Using a targeted approach, we proceeded to the fine mapping analysis of the unique CpGs (Fig 4) surrounding the previously identified *ANGPTL2* methylation site covered by the selected probe cg09427311.

**Fig 5:**
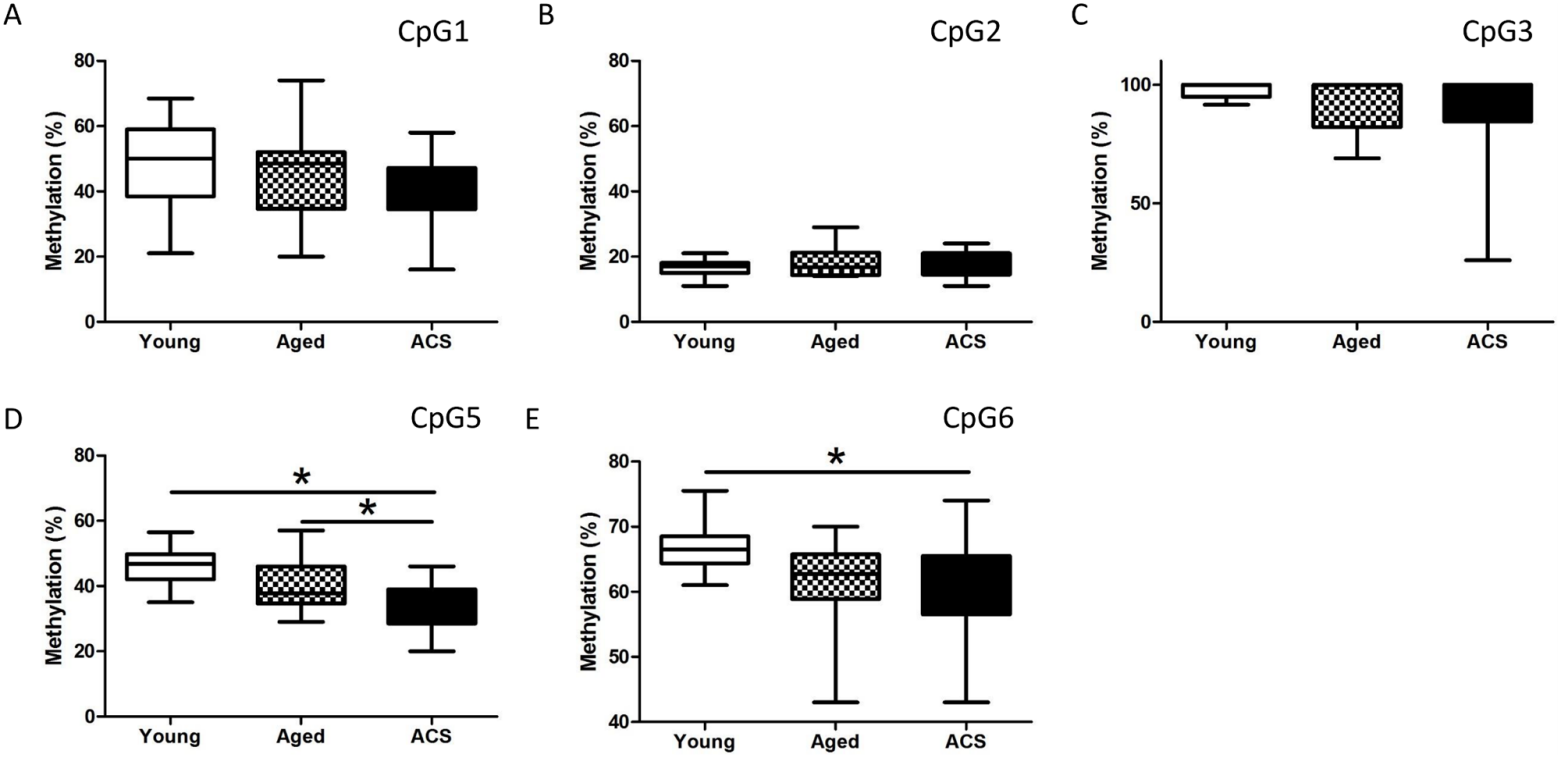
Hypomethylation of CpG5 and CpG6 in post-ACS patients. Methylation percentage of the methylation sites (A) CpG1, (B) CpG2, (C) CpG3, (D) CpG5 and (E) CpG6, previously identified in Fig 4. DNA was isolated from leukocytes of post-ACS patients (n = 21), age-matched (n = 12) and young (n = 14) healthy controls. Box and whiskers plot of (n) participants, *: p<0.05 (Kruskal-Wallis test).

Of the 6 CpGs found within the 344 bp region amplified by specific primers, 2 CpGs (CpG5 and CpG6) show differential methylation between groups (Fig 5). Methylation of CpG5 was significantly decreased in post-ACS patients (34.7 ± 1.4%; p < 0.05, n = 21) when compared to young (45.8 ± 1.5%, n = 14) and aged-matched (40.6 ± 2.3%, n = 12) control groups. However, no difference was observed between young and age-matched control groups (Fig 5D). Compared to young controls (66.6 ± 0.9%, n = 14), methylation of CpG6 was also lower in post-ACS (60.4 ± 1.5%; p < 0.05, n = 21), but no significant difference was observed with age-matched controls (61.4 ± 2.0%, n = 12). Again, methylation of CpG6 was similar between both control groups (Fig 5E).

These results suggest that CpG5 methylation is sensitive to the disease state since a significant hypomethylation is detected in post-ACS patients when compared to both healthy groups. Conversely, it is unclear which factor regulates CpG6 methylation due to the lack of discrepancy between the post-ACS group and the age-matched controls or between the young and age-matched controls.

On the other hand, no significant variations in the methylation levels among groups were detected for CpG1, CpG2 and CpG3 (Fig 5A–5C). The remaining CpG4 could not be analyzed due to a limitation of this assay: the EpiTYPER technology relies on a mass spectroscopy analysis of CpG-containing DNA fragments by uracil-specific cleavage and the CpG4 fragment was too small to be reliably detected by mass spectrometry (data not shown).

### *ANGPTL2* methylation is correlated with CRP levels

**Fig 6:**
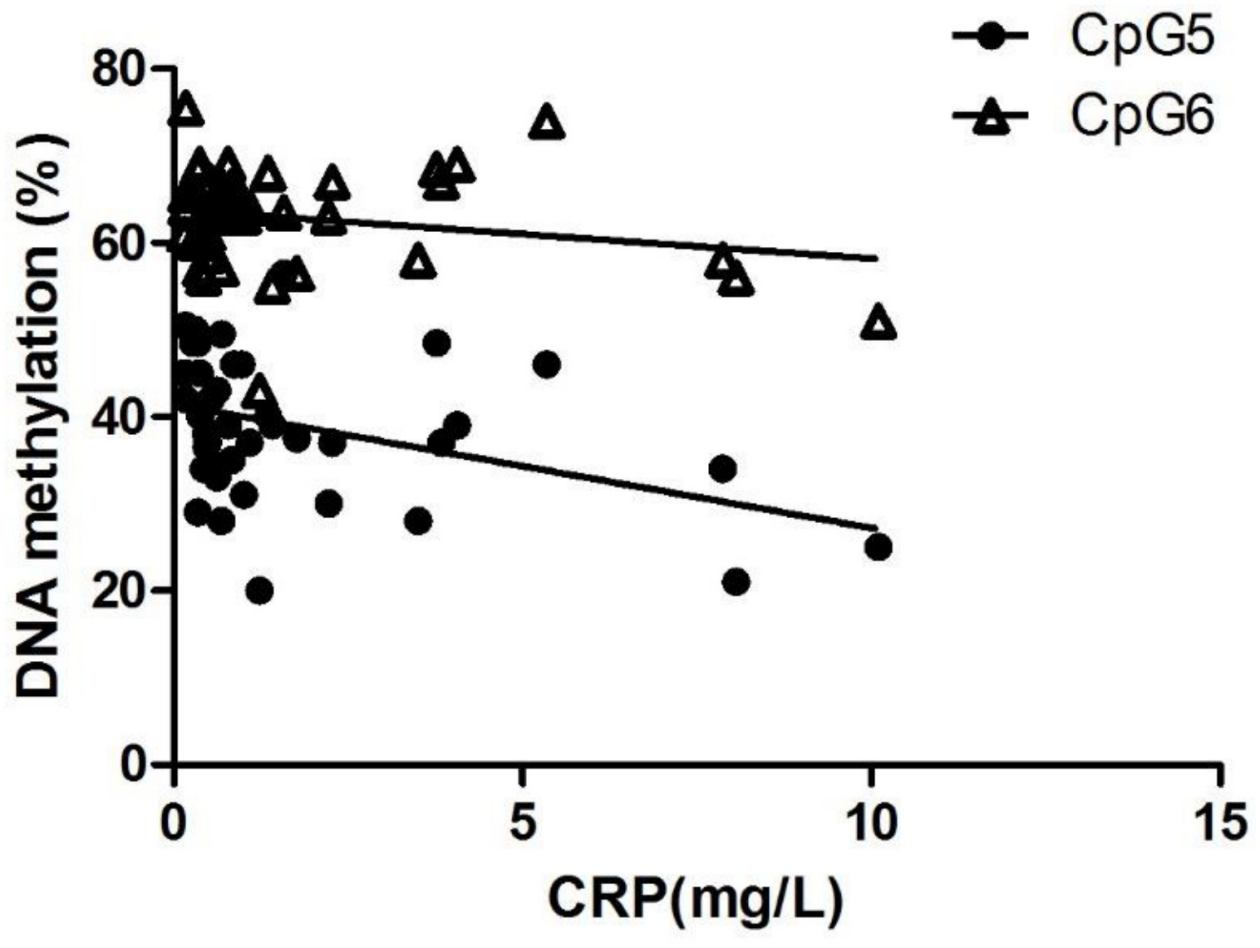
CpG5 methylation is inversely correlated with CRP concentration. Negative correlation between plasma CRP concentrations and CpG5 (p = 0.0096, r = -0.395, n = 42) and CpG6 (p = 0.1731, r = -0.214, n = 42) methylation in all participants (n = 14 young healthy controls, n = 9 age-matched healthy controls, n = 19 post-ACS patients).

Interestingly, CpG5, but not CpG6 methylation negatively correlates (p<0.05) with circulating CRP levels when all participants were considered (Fig 6), suggesting that *ANGPTL2* hypomethylation is associated with high levels of CRP, establishing a link between *ANGPTL2* methylation and inflammation. For this reason, only CpG5 was considered for further analysis.

### *In vitro* methylation decreases *ANGPTL2* promoter activity

**Fig 7:**
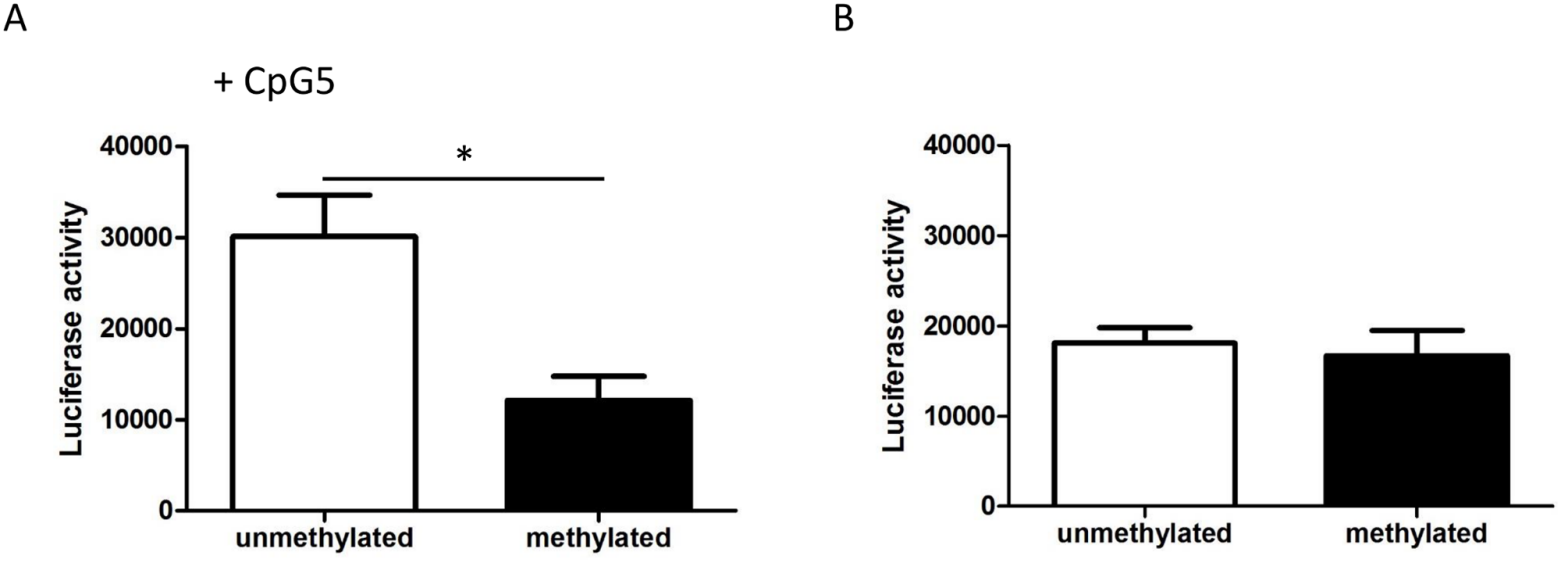
*In vitro* methylation of *ANGPTL2* decreases promoter activity. *In vitro* methylation of *ANGPTL2* target region containing CpG5 inhibited transcriptional activity, as measured by a luciferase reporter assay. Luciferase activity of methylated (M.SssI treated) and unmethylated constructs (A) containing the CpG5 site or (B) without. The assay was repeated 3 times and data are mean ± SEM. *: p<0.05 versus unmethylated (Unpaired t-test).

To assess the impact of CpG5 methylation on ANGPTL2 expression, we used an *in vitro* methylation luciferase assay as previously described [54–56]. A 32 bp promoter sequence containing CpG5 was inserted into the pCpGfree-basic vector, upstream of a hEF-1α CpG-free promoter. This construct was then methylated *in vitro* and subsequently transfected in HEK293 cells where luciferase activity was measured as an indicator of promoter activity. *In vitro* methylation of the construct containing the *ANGPTL2* promoter fragment significantly reduced promoter activity as shown by a decrease of 60% (p < 0.05) in luciferase activity (Fig 7A). Conversely, methylation of the vector lacking the CpG5 sequence did not alter promoter activity (Fig 7B). These results suggest a potential molecular regulatory role of CpG5 methylation on ANGPTL2 expression.

## Discussion

In this study, we show for the first time that *ANGPTL2* methylation pattern varies in post-ACS patients and that this methylation pattern is independent of aging. During the course of the study we also identified a novel regulatory region in the *ANGPTL2* promoter, the CpG5, which is hypomethylated in association with the pro-inflammatory environment in these patients. We found that *in vitro* methylation of CpG5 induced a lower transcriptional activity. Therefore, differential CpG5 methylation pattern may identify patients at risk of a first cardiovascular event.

Previous studies suggest that ANGPTL2 expression is regulated by DNA methylation. For example, hypermethylation of CpGs located in *ANGPTL2* gene have been reported in various ovarian cancer cell lines, where *ANGPTL2* is silenced [34] and in bone marrow samples from patients suffering from primary myelodysplastic syndrome [35]. In contrast, hypomethylation of *ANGPTL2* promoter has been observed in human osteosarcoma cell lines, proportionally with the increase in ANGPTL2 expression and progression of the disease when these cells were injected in mice [36]. In our study, we observed a decrease in *ANGPTL2* methylation in the CpG5 region in post-ACS patients. In our hands, human leukocytes do not produce detectable levels of ANGPTL2 and mRNA levels were at the low detection limit (data not shown). Nonetheless, our data suggest that methylation of CpG may represent a mechanism of regulation that could in part account for the elevated circulating levels of ANGPTL2 in these patients. Indeed, we have demonstrated that two specific methylation sites, CpG5 and CpG6, are less methylated in diseased patients when compared to young healthy controls. However, only CpG5 significantly differed from the age-matched control group and significantly correlated with CRP levels, suggesting that potential methylation sites are differently sensitive to various stimuli such as age and the disease state. This is also supported by the observation that methylation in surrounding CpGs (CpG1-3) do not significantly vary in any of our groups. The amplitudes of the changes in methylation levels observed in our study (>5 to 10%) are in line with what is typically observed in studies conducted on white blood cells in a pro-inflammatory context [57, 58]. In addition, a previously published study conducted on cord blood cells in association with maternal obesity revealed that *ANGPTL2* methylation differed by less than 5% across body mass index categories [59]. Aberrant methylation patterns have been extensively studied in the context on chronic inflammatory diseases. In cancer, global DNA methylation measured from the blood can be used as a biomarker for cancer risk [60]. In CVD, low-density lipoproteins exert their effect on endothelial cells through changes in DNA methylation [61, 62] and atherosclerosis is characterized by a global state of hypomethylation [63]. Therefore, it could be speculated that changes in methylation patterns could reflect the health status of the patients and be much more specific of the pathology involved compared to the circulating levels of the protein.

Indeed, as previously mentioned, our group [4, 20] and others [5, 12, 13] have demonstrated that circulating ANGPTL2 concentration is increased in a pro-inflammatory context in a proportional manner to the severity of the disease. In our study, only a small difference was observed in Angplt2 plasma concentrations of post-ACS patients when compared to age-matched healthy controls, reflecting a lesser or shorter cumulative inflammatory burden compared to that of patients with known CVD and a longer history of cardiovascular events [20]. We can hypothesize that in the presence of a more severe inflammatory environment such as in patients with established CVD, which would be highlighted by higher ANGPTL2 levels, changes in *ANGPTL2* methylation may become detectable in other CpGs. Such graded methylation has been previously reported in cancer: methylation of *ANGPTL2* varies proportionally with tumour metastasis [36].

ANGPTL2 is often associated with markers of inflammation such as CRP [64, 65], IL-6 and TNF-α [4, 66] and although it is not always clear which comes first, it is acknowledged that ANGPTL2 participates in a pro-inflammatory loop by being sensitive to inflammation and in turn, further promotes inflammatory pathways. In our study, *ANGPTL2* methylation at CpG5 is inversely correlated with CRP. Furthermore, CpG5 and CpG6 methylation is decreased in leukocytes from post-ACS patients who also happen to have higher levels of circulating ANGPTL2 and CRP; this suggests that a pro-inflammatory environment may favour the production of ANGPTL2 in part by decreasing DNA methylation in the relevant producing cells. An interesting finding by Sasaki and al. [26] indeed states that ANGPTL2 can act in an autocrine manner. Their work shows that treatment of macrophage-like cells with ANGPTL2 increases its own expression in a dose-dependent manner [26]. Taking these results together, we can hypothesize that in addition of inflammation *per se*, ANGPTL2 could induce its own expression through a DNA methylation mechanism.

### Limitations of the study

The present study allows us to observe the methylation changes of *ANGPTL2* in a context of mild inflammatory stress in optimally treated post-ACS patients. Our group has previously demonstrated that patients with chronic documented coronary artery disease exhibit greater signs of inflammation through slightly higher circulating ANGPTL2 (3.35 ± 0.67 ng/mL for post-ACS *versus* 5.74 ± 0.75 ng/mL for CAD) [20]. It would be interesting to study *ANGPTL2* methylation under such conditions.

Following the exploratory experiment aiming to identify methylation candidates, we narrowed our target CpGs down to 6 potential regulatory CpGs. Hence, DNA methylation quantification approaches covering broader *ANGPTL2*-related CpGs and regulatory regions should be considered, especially when considering patients with longer history of risk factors and longer history of cardiovascular events. Therefore, other regulatory methylation sites previously characterized by others [34–36] in different pathological contexts could be included in future studies enrolling patients with CVD. This would allow us to determine how these epigenetic marks can differ when comparing various types of inflammatory diseases. Based on the limited literature on the subject of *ANGPTL2* methylation, we observe contrasting results; in cancer cells, researchers have reported a decrease in *ANGPTL2* methylation resulting in an increased ANGPTL2 expression with the progression of the disease [36] while others [34, 35] have shown the opposite. These illustrate how epigenetic mechanisms can vary within the same type of disease by taking into consideration the cell type.

Leukocytes represent an accessible and reliable way to obtain DNA with little discomfort to the patient. However, it is a mixed population of cells in which methylation patterns may be different [67]. Although immune cells express ANGPTL2 [6, 23, 24] and are likely not the main contributor to the circulating pool of ANGPTL2 [5], further studies should isolate the mixed leukocytes population.

In conclusion, reduced leukocyte DNA methylation in the promoter region of *ANGPTL2* is associated with the pro-inflammatory environment that characterizes post-ACS patients differently from age-matched healthy controls. Importantly, our data suggest that methylation of different CpGs in *ANGPTL2* may prove to be a reliable biomarker of coronary disease. Replication of our study in a wider range of CpGs in patients with different combination of risk factors for CVD and a history of cardiovascular events should validate the usefulness of methylation patterns in *ANGPTL2* as a biomarker for a better risk assessment of future cardiovascular events.
